# Advancing affective stimuli databases: challenges and solutions

**DOI:** 10.3389/fpsyg.2025.1589612

**Published:** 2025-09-18

**Authors:** Marina M. Gerges, Danila Shelepenkov, Vladimir Kosonogov

**Affiliations:** ^1^Institute for Cognitive Neuroscience, HSE University, Moscow, Russia; ^2^Affective Psychophysiology Laboratory, Institute of Health Psychology, HSE University, Saint Petersburg, Russia

**Keywords:** affective science, databases, stimuli, psychophysiological research, affective stimulus databases

## Abstract

Affective stimulus databases are integral elements in psychological and neuroscientific research, enabling the controlled induction of emotional states. However, despite significant progress, existing databases face methodological limitations that interfere with cross- study comparability and reproducibility. This review thoroughly examines modern affective stimulus databases across visual, auditory, textual, and multimodal domains, presenting their positive attributes and deficiencies. Key challenges include variability in stimulus standardization, inconsistencies in validation procedures, cultural specificity, and reliance on either categorical or dimensional emotion assessment methods. Additionally, issues related to stimulus diversity, duration control, and ecological validity further complicate the interpretation of results in psychophysiological studies. To address these challenges, we propose strategies for improving future databases, including the integration of standardized evaluation methodologies, the expansion of multimodal and culturally diverse stimuli, and the implementation of advanced technological solutions such as virtual reality and machine learning. Improving the structure of databases and maintaining consistent methodologies will increase the reliability and applicability of emotion research, ultimately contributing to a more comprehensive understanding of affective processes across different fields.

## 1 Introduction

To date, numerous researchers have sought to understand emotions, their origins, physiological correlations, and how they shape human behavior. Emotions are complex phenomena, encompassing both conscious and unconscious components, modulated by a variety of social, cultural, cognitive, and physiological factors (Izard, 2009; Barrett, 2017). The definition of emotion remains a subject of debate, with some theories emphasizing discrete categories (Ekman, 1992), such as anger or fear, and others advocating for dimensional models (Russell, 1980), which conceptualize emotion along continuous axes like valence and arousal. This distinction between models also shapes how emotional stimuli are developed and validated in experimental databases. Affective neuroscience continues to investigate the neural substrates underlying emotions (Lindquist et al., 2012; Kober et al., 2008). However, the emotion study relies on diverse protocols of induction, from visual and auditory stimuli to complex social scenarios. This heterogeneity often obscures findings and impedes progress in the field (Lang and Bradley, 2010; Schaefer et al., 2010). This review critically evaluates how this imbalance affects methodological consistency and cross-study comparability. We provide an overview of stimulus databases used to induce emotions. Our aim is not to outline existing databases but rather to critically evaluate them, highlighting their strengths and limitations. Hence, we propose several ideas for improving the organization and standardization of future databases to enhance the quality of emotion research. By addressing the current limitations of affective stimuli databases and advocating for standardized and multimodal approaches, this review aims to contribute to more replicable, and generalizable findings in the study of emotions (Bradley and Lang, 2007; Kurdi et al., 2017).

## 2 Overview of emotional stimulus databases

Affective stimulus databases were developed to standardize emotion induction protocols. Some early efforts focused on visual (IAPS) (Lang et al., 1997) and auditory stimuli (IADS) (Bradley and Lang, 1999). Over subsequent decades, databases diversified to include facial expressions (e.g., NimStim) (Tottenham et al., 2009), video clips (DEVO) (Ack Baraly et al., 2020), and culturally specific stimuli (e.g., EmoMadrid) (Carretié et al., 2019). By 2020, the field had expanded to encompass multimodal and virtual reality (VR) stimuli, reflecting technological advancements and a growing emphasis on ecological validity.

A landmark effort to consolidate these resources is the KAPODI database (Diconne et al., 2022), which cataloged 35 auditory, 117 facial, 35 pictorial, 43 video, 89 textual, and 45 mixed-modality affective databases published between 1963 and 2020. KAPODI provides metadata for the year, sample sizes, descriptions, and validation methodologies, serving as a critical resource for researchers navigating the fragmented landscape of affective stimuli.

Post-2020 advancements have focused on addressing gaps in cultural specificity, multimodal integration, and ecological validity. Among these developments, The Empathy for Pain Stimuli System (Meng et al., 2023) uses visual and contextual cues to depict pain-inducing scenarios, validated for eliciting empathic responses. The ESISCA (Zhang et al., 2023) provides 274 images of social interactions conveying happiness, anger, sadness, fear, disgust, and neutral emotions for studying emotion recognition and empathy in social contexts. The SocialVidStim (Tully et al., 2024) offers video stimuli of social evaluations (e.g., praise, criticism), validated for ecological relevance. Additionally, the luVRe (Schöne et al., 2023) provides immersive 3D/360° stimuli for VR, while An Open-Access Database of Video Stimuli for Action Observation Research supports neuroimaging studies of motor cognition (Georgiev et al., 2024). These databases emphasize open-access principles, cultural sensitivity, and technological innovation.

## 3 Classification of emotional stimuli

There are several approaches for classifying emotional stimuli; however, we argue that the most fundamental distinction is based on modality. Different modalities evoke distinct physiological and psychological responses, making this classification particularly relevant for affective neuroscience and psychology (Schirmer and Adolphs, 2017).

Visual stimuli, particularly static images, are widely used in affective research due to their ease of control, reproducibility, and standardization (Lang et al., 1997). Prominent databases such as the International Affective Picture System (IAPS) and OASIS provide a broad range of affective ratings (Kurdi et al., 2017), though they primarily used the dimensional model of emotion. Among image-based databases, several specialized resources stand out. For example, GAPED focuses on fear-related stimuli (Dan-Glauser and Scherer, 2011), NAPS provides culturally neutral content (Marchewka et al., 2014), and ESISCA captures culturally informed social interactions (Zhang et al., 2023), thereby broadening the scope of affective visual material.

A notable subcategory within visual stimuli includes databases of emotional facial expressions, such as KDEF (Lundqvist et al., 2015) and Ekman’s series (Ekman and Friesen, 1976). These databases typically follow the discrete model of emotion, focusing on basic emotions and their corresponding facial expressions.

Dynamic visual stimuli—such as video clips and virtual reality (VR) content—offer enhanced ecological validity compared to static images, as they better approximate real-life emotional experiences. However, they introduce challenges in standardization, including variability in video duration, resolution, and audiovisual quality. Many of these databases utilize videoclips from unfamiliar sources (Ack Baraly et al., 2020) or well-known films. Notable examples include the Emotional Film Clips database (Gross and Levenson, 1995) and FilmStim (Schaefer et al., 2010), both of which offer carefully curated and validated video excerpts for emotion elicitation.

Recent advances have led to the development of more socially and contextually rich stimuli. For instance, SocialVidStim (Tully et al., 2024) could be fruitful for studying cognitive processes related to social interaction, while luVRe (Schöne et al., 2023) offers immersive 3D/360° VR environments. VR-based affective databases such as PanoEmo (Kosonogov et al., 2024), AVDOS-VR (Gnacek et al., 2024), and IAVRS (Mancuso et al., 2024) provide standardized emotional content within immersive virtual settings. These tools aim to increase emotional realism and participant engagement.

Not Only Visual Stimuli but also auditory ones, including music, speech, and environmental sounds, effectively induce emotions due to their strong link to autonomic responses (Bradley and Lang, 1999). These stimuli can be either nonverbal or verbal. Nonverbal auditory stimuli include emotionally expressive sounds, such as those found in the International Affective Digitized Sounds (IADS), emotionally annotated music tracks provided by EmoMusic (Soleymani et al., 2013), and domain-specific classifications of musical emotions featured in the Geneva Emotional Music Scale (GEMS) (Zentner et al., 2008). Verbal auditory stimuli encompass speech, pseudospeech, and vocal expressions. These types of stimuli often require linguistic and cultural adaptation to ensure emotional relevance and accuracy (Pell et al., 2015). Currently, most verbal auditory databases are available in a limited number of languages. However, there are notable exceptions, such as the VENEC and Vocally Expressed Emotions datasets, which aim for broader applicability. Notable databases include Brussels Mood Inductive Audio Stories (Bertels et al., 2014) for mood elicitation, as well as culturally specific sets like Chinese Vocal Emotional Stimuli (Liu and Pell, 2012) and the Italian Emotional Speech Database (Costantini et al., 2014).

Text-based stimuli provide an alternative method for emotion induction, particularly valuable in semantic and cognitive research. However, their effectiveness largely depends on participants’ linguistic proficiency and cultural background (Kanske and Kotz, 2010). Most text-based databases rely on dimensional models of emotion, primarily focusing on affective dimensions such as valence and arousal, occasionally incorporating additional dimensions. A widely used example is the Affective Norms for English Words (ANEW) database (Bradley and Lang, 1999), along with its various adaptations, such as the Spanish version (Redondo et al., 2007). While textual stimuli offer high experimental control and ease of implementation, they lack the sensory and contextual richness inherent in visual and auditory modalities, which can limit the depth and immediacy of emotional responses they elicit.

Multimodal stimuli combine multiple sensory inputs, such as images and sounds, to enhance ecological validity (Baumgartner et al., 2006). Although relatively few studies directly compared the effectiveness of emotional elicitation across different modalities, multimodal stimuli hold significant promise. They evoke stronger autonomic responses—such as skin conductance, pupil dilation, and heart rate changes—which are widely used as physiological indicators of emotional arousal and engagement (Bradley et al., 2001; Bradley et al., 2008). These responses correlate with both valence and arousal dimensions and contribute to validating the emotional impact of stimuli (Baumgartner et al., 2006; Fan et al., 2020). Additionally, multimodal input activates broader neural networks. For instance, increased alpha power during audiovisual conditions suggests deeper emotional processing (Fan et al., 2020). Baumgartner et al. (2006) found that combining music with emotional images enhanced emotional responses across self-report and physiological measures.

Moreover, multimodal stimuli often possess greater ecological validity, simulating real-world scenarios more effectively. This may improve the generalizability of findings to everyday experiences (Brück et al., 2011). However, multimodal results can sometimes be less stable than those elicited by simpler stimuli. Static images may offer superior control and reproducibility, making them ideal for eliciting certain emotions under tightly controlled conditions (Schmidt and Trainor, 2001), controlling over participants’ focus, and reducing attentional drift (Lang et al., 1997). Conversely, prolonged exposure to dynamic or multimodal stimuli may be better suited to study sustained emotional responses, empathy, or narrative engagement (Schaefer et al., 2010). While multimodal stimuli offer greater realism, they introduce methodological complexities, including the need to control sensory integration effects and participant variability in immersive experience.

About half of all databases and most of the image databases (that do not include facial expressions) rely on the dimensional model of emotion, which conceptualizes affective experience along two continuous axes: valence describes the extent to which an emotion is positive or negative (ranging from unpleasant to pleasant), whereas arousal refers to its intensity (ranging from calm to excited) (Russell, 1980). This framework, known as the dimensional model of affect, is widely adopted but often excludes other models such as discrete emotion categories (e.g., anger, fear, sadness), potentially limiting cross-paradigm comparisons. In some cases, a third dimension—dominance (control vs. submission)—is also included, as in the ANEW word database. Some other dimensions can be added depending on the goal of a study, like social load (Kosonogov et al., 2020) and craving (Suissa-Rocheleau et al., 2019) or semantic content (Itkes et al., 2019).

However, few databases encompass both dimensional and categorical models; that is, they represent the model that supposes that each discrete affective state should lie along valence and arousal dimensions (Russell, 1980). Notable exceptions include DEAP (Skaramagkas et al., 2023), which combines valence-arousal ratings with discrete emotion categories of musical videos with physiological data, and luVRe (Schöne et al., 2023), which offers immersive VR stimuli rated along both dimensions and discrete labels.

Other relevant variables such as ecological validity and visual complexity can also influence emotional responses and are increasingly considered in newer databases. These factors, while not part of traditional models, contribute to how stimuli can be perceived and processed. Terminology within this domain also varies across studies—for instance, “emotion elicitation,” “emotion recognition,” and “emotion perception” are often used interchangeably, though they refer to distinct processes (Lindquist et al., 2012; Schirmer and Adolphs, 2017). Greater precision and consistency in these terms would help reduce confusion and support better methodological alignment across studies.

## 4 Methodological considerations

Meta-analyses face challenges due to the variability in emotion induction methods across studies. Kirby and Robinson (2017) noted diverse protocols, including music, memory recall, facial expressions, words, and imagery. Similarly, Lindquist et al. (2012) combined studies using film clips, facial stimuli, and imagery, while other meta-analyses (Murphy et al., 2003; Kober et al., 2008; Vytal and Hamann, 2010) integrated data from mixed protocols such as visual, auditory, and memory-based methods. Despite their value, meta-analyses assume comparability among methods, though emotional perception can vary due to the stimulus modality (Schirmer and Adolphs, 2017; Fan et al., 2020; Murphy et al., 2003). This highlights the need for a nuanced approach that accounts for modality effects.

Methodological inconsistencies across affective databases often stem from the emotion model adopted. As we already mentioned, dimensional approaches, which dominate current databases, typically assess valence and arousal using continuous scales such as the Self-Assessment Manikin (Polo et al., 2024). Categorical models, by contrast, classify emotions into discrete categories, such as basic emotions or context-dependent states like distress or comfort. Each model shapes how emotions are measured, validated, and interpreted: dimensional scales allow for subtle gradations, whereas categorical labels may offer more intuitive clarity but risk oversimplifying affective complexity (Salikova and Kosonogov, 2025). Most databases rely exclusively on one framework, which may limit their adaptability across study designs and research questions.

The overall quality of affective databases also depends on their normative data and validation processes. While early databases often used large and diverse samples (Branco et al., 2023), others rely on smaller, culturally homogenous groups, reducing generalizability. In addition, the choice of rating instrument—whether Likert scales (and the number of points), visual analogue scales, or SAM—affects measurement sensitivity and interpretability. Researchers should also consider confounds such as order effects (Kosonogov, 2020), participant fatigue (Singh et al., 2025), and cultural biases (Morris, 1995), all of which can impact the reliability and replicability of emotional data. In addition, ethical concerns and cultural specificity present further challenges in emotion research. The availability of affective stimuli that are both standardized and ethically approved is crucial. In the early stages of database development, professional photographers and videographers were often commissioned to produce stimuli, especially for content that was difficult to access (like violent or erotical material). Today, many databases rely on open-access images and films or provide web links to stimuli; however, these links may become obsolete over time as noted by Li et al. (2017). Some affective stimuli, particularly those from older databases, may no longer align with the experiences or cultural contexts of newer generations, potentially affecting the emotional responses they elicit.

Furthermore, because emotional responses can vary significantly across cultural contexts, a stimulus that is validated in one cultural setting may not evoke the same response in another. Cross-cultural research reveals that emotional responses are strongly influenced by physical and social environments. Jonauskaite et al. (2019) showed that participants in less sunny regions across 55 countries were more likely to associate yellow with joy, while Huwaë and Schaafsma (2018) found that individuals from collectivistic cultures suppress both positive and negative emotions more. Researchers should therefore validate foreign databases in their local context or develop culture-specific stimuli for the target population.

The application of emotional stimulus databases in psychophysiological research requires careful consideration. Static stimuli (e.g., pictures, words) allow precise control, whereas dynamic stimuli (e.g., videos, spoken or written texts, VR) evoke more complex nervous processes, complicating physiological measurements. Many databases have been successfully used in studies employing fMRI (Caria, 2020), EEG (Hajcak and Dennis, 2009), MEG (Styliadis et al., 2014), skin conductance (D’Hondt et al., 2010), heart rate (Bradley et al., 2001), and EMG (Baglioni et al., 2010). Additionally, abrupt sounds have been used to elicit the startle blink reflex, reflecting valence (Vrana et al., 1988; Kosonogov et al., 2016), while the eSEE-d dataset captures eye movements during emotion-evoking videos (Kreibig, 2010).

## 5 Limitations of current databases

Despite significant advances in developing affective stimulus databases, several limitations persist, affecting their applicability in psychophysiological research and affective science. One of the primary challenges is the *lack of standardization* across databases, making it difficult to compare findings across studies (Bradley and Lang, 2007). Differences in stimulus quantity, rating methodologies, and validation procedures can introduce inconsistencies that affect the reliability and reproducibility of research (Mollahosseini et al., 2019). Additionally, while some databases have been validated using large and diverse participant samples, others rely on smaller, culturally homogenous groups, limiting the generalizability of findings (Dan-Glauser and Scherer, 2011). At the same time, culturally specific databases are essential for capturing population-relevant emotional responses. We acknowledge this tension and suggest that future research should balance both goals by either validating existing databases across multiple cultural contexts or by developing parallel culturally grounded versions using shared protocols. To improve standardization more broadly, we recommend the use of common rating instruments (e.g., Self-Assessment Manikin or 9-point Likert scales), clear reporting of physical stimulus properties (e.g., duration, brightness, resolution), and inclusion of participant demographic metadata. Standardized documentation formats and open-access repositories would further enhance reproducibility and cross-study comparability. Addressing these limitations is essential for improving the robustness and comparability of studies employing affective stimuli. There is considerable variability in *the number of stimuli* provided across studies. Although many researchers claim to use large databases, the actual stimulus count often remains inconsistent and insufficiently standardized. For example, the IAPS (Lang et al., 1997) offers a well-balanced set of stimuli across emotional categories, whereas smaller databases frequently lack sufficient stimuli per category, limiting their utility for robust experimental designs. This variability can lead to difficulties when researchers attempt to compile comprehensive stimulus sets that adequately represent a broad range of emotional states. As a positive example, the OASIS (Kurdi et al., 2017) provides 900 public domain images with normative ratings on valence and arousal, effectively addressing some of the limitations observed in earlier databases.

*The number of raters* for validating affective stimuli is often limited. For instance, while large-scale datasets like AffectNet (Mollahosseini et al., 2019) use online crowdsourcing to gather normative data, each image is typically annotated by only one rater (with a small subset receiving a second annotation for reliability). In contrast, some dynamic stimuli databases rely on samples of fewer than 50 participants, which may limit the generalizability of the ratings. Moreover, inconsistent stimulus durations can introduce variability in physiological responses (e.g., heart rate and skin conductance), further complicating result interpretation (Bradley et al., 2001).

Most affective databases rely exclusively on a single *measurement* approach—either dimensional (e.g., valence and arousal) or categorical (e.g., basic emotions)—which restricts the scope of emotional assessment. While the DEAP database (Koelstra et al., 2012) effectively combines physiological data with dimensional ratings, many databases depend solely on one type of assessment. For example, the NimStim facial expression set (Tottenham et al., 2009) relies exclusively on categorical labels obtained from a relatively small group of raters, limiting its ability to capture the full complexity of affective experiences. Because emotional responses are inherently multidimensional, the absence of a comprehensive, integrated approach can hinder cross-study comparisons and reduce the interpretability of findings (De Cesarei and Codispoti, 2010).

*Physical characteristics* such as brightness, loudness, complexity, and representational format (symbolic vs. photographic) vary significantly across affective databases, potentially introducing unintended confounds. While some databases, like EmoMadrid (Carretié et al., 2019), control for brightness and contrast, others contain substantial variability that may influence attentional engagement and neural processing. De Cesarei and Codispoti (2010) found that even factors like image size can affect psychophysiological responses, while Nejati (2021) demonstrated that 3D stimuli impose a higher cognitive load than 2D images, increasing pupil dilation and saccadic eye movements. These findings underscore the importance of standardizing physical properties within affective databases to minimize unintended influences on emotional and physiological measurements.

From a psychophysiological perspective, it is ideal for stimuli to be of *uniform duration* to ensure that evoked responses such as heart rate, skin conductance, and neural activity are directly comparable across conditions. However, many affective video databases either include clips of varying durations or, when they standardize duration, provide too few examples to support reliable comparisons across different emotions. For example, the DEVO includes video clips ranging in length from 3 to 15 s (Ack Baraly et al., 2020), complicating the interpretation of physiological data due to inconsistencies in exposure times. In contrast, the Database of Visual Affective Stimuli (Li et al., 2022) standardizes all clips to a single 3 s duration, providing greater control over stimulus presentation. However, while such databases improve methodological consistency, they may not include sufficiently diverse clips for studies requiring detailed comparisons of specific emotions, such as distinguishing psychophysiological responses to sadness and anger. Expanding standardized databases to include a wider range of emotional content while maintaining strict temporal control remains a key challenge in affective research. A new study by Kosonogov et al. (2025) introduces a database of one-minute silent video clips with valence and arousal ratings, providing 160 affective videos. This development represents a step toward standardizing and ensuring the diversity of clips needed for more nuanced emotional research. Key methodological considerations for improving database development are summarized in Table 1.

**Table 1:** Key methodological considerations for affective databases.

Methodological consideration	Recommendation	Supporting references	Relevant databases
Number of stimuli per category	Affective categories (negative, neutral, positive) should each include at least 10 stimuli to provide balanced representation and capture emotional variability across participants.	Schindler et al. (2017), Mikels et al. (2005), Bänziger et al. (2012), and Pant et al. (2023)	IAPS, OASIS, ANEW, GEMEP, PhyMER (Bänziger et al., 2012; Pant et al., 2023)
Number of raters per stimulus	Each stimulus should be rated by at least 100 participants to ensure robust and reliable normative data. Subsampling can be used for large databases, ensuring diversity in ratings.	Bradley and Lang (2007), Kurdi et al. (2017), Livingstone and Russo (2018), and Ma et al. (2015)	AffectNet, RAVDESS (Livingstone and Russo, 2018), Chicago Face Database (CFD) (Ma et al., 2015)
Dimensional vs. categorical models	Databases should integrate both dimensional (e.g., valence, arousal) and categorical (e.g., basic emotions) rating systems for more comprehensive emotion measurement.	Libkuman et al. (2007) and Morgan (2019)	IAPS (Libkuman et al., 2007), ANEW, Morgan Emotional Speech Set (Morgan, 2019)
Standardization of stimuli	Standardize non-affective properties, including visual (size, resolution), auditory (volume, pitch), and text (length, segmentation), to minimize confounds and enhance stimulus control.	Lakens et al. (2013), Jaquet et al. (2014), and de Gelder and Van den Stock (2011)	IAPS, OASIS, DEAP, BEAST (de Gelder and Van den Stock, 2011)
Cultural specificity	Future databases should either include culturally diverse samples, or be validated across multiple cultural contexts to ensure generalizability and ecological validity of emotional responses.	Pham and Sanchez (2019) and Bradley et al. (2008)	EmoMadrid, GAPED, OASIS, KDEF

## 6 Future directions

To address these limitations and move toward an ideal affective database, several improvements are proposed.

First, each subset of stimuli should consist of at least 10 items for each affective category (e.g., negative, neutral, positive) to ensure balanced representation. Using 10 stimuli per emotion ensures a sufficient variety of facial expressions to represent each emotion across different intensities and individual differences. This number strikes a balance between maintaining statistical power and minimizing participant fatigue. It aligns with the standard practice in emotion perception research, where studies often utilize between 5 and 10 stimuli per emotion to achieve reliable and generalizable results without overburdening participants (Mikels et al., 2005; Pham and Sanchez, 2019). This can help psychophysiologists to find a sufficient number of stimuli to provoke reliable reactions, since they depend on personal experience of subjects. In psychological studies, more stimuli may be presented, if they do not suppose baseline intervals, resting states, etc. However, in other EEG paradigms, significantly larger sets of stimuli or trials are typically required to obtain reliable results. For example, emotion recognition datasets used in machine learning, such as SEED-VII (Jiang et al., 2024), include only 12 video clips per emotion—a relatively limited amount when compared to methodological recommendations for event-related potentials (ERPs), another widely used EEG-based approach. Depending on the specific ERP component, the recommended number of trials ranges from a minimum of 8 for detecting error-related negativity (ERN) (Boudewyn et al., 2018) to approximately 50 trials for reliable motor-related responses (Borràs et al., 2022). Second, an adequate number of raters—ideally in the hundreds—should assess each stimulus to ensure robust and reliable normative data (Bradley and Lang, 2007). Increasing the number of raters improves the reliability of ratings by minimizing the influence of outliers or biases and allows for statistical power to analyze subgroup differences (Kurdi et al., 2017). For large databases, this is achieved by having different subsample rates for distinct stimulus subsets. In IAPS, thousands of images are divided into smaller sets (e.g., 100 images per subset), each rated by a unique group (Bradley et al., 2008). Similarly, ANEW, which includes hundreds of words, assigns smaller groups (e.g., 50 words per group) to ensure adequate ratings while distributing the workload efficiently (Bradley and Lang, 1999). Such strategies enhance representativeness, variability, and the reliability of normative data.

Third, future databases would benefit from the integration of both dimensional and categorical rating approaches. We suppose that such integration could contribute to the long-standing debate between discrete and dimensional models of emotion. A broad data collection of both discrete and dimensional ratings could help reveal some relationships between different variables used in both approaches and possibly reconcile them. In any case, tagging affective stimuli in both approaches may help researchers to test some hypotheses from different angles. One technical solution may involve assigning different groups of raters to distinct parts of the evaluation (for example, one group using dimensional scales and another using categorical labels) and indicating the method used in the database description. Another possibility would be to ask subjects to rate half of stimuli using one approach and another half with another approach.

Finally, it is critical to control and equalize all non-affective properties of stimuli. For visual content, this includes standardizing size, resolution, brightness, edge density, and duration; for auditory stimuli, maintaining consistent volume and pitch; and for text, ensuring uniform length and segmentation. While full standardization may not be feasible, minimizing confounds ensures responses are primarily driven by affective content. Brighter images tend to be rated as more positive (Lakens et al., 2013), higher visual complexity enhances occipital event-related potentials (Schmidt and Trainor, 2001), and emotional scenes show greater spectral density in low spatial frequency bands (Delplanque et al., 2007). In audio, pitch variations significantly affect perceived pleasantness and arousal (Jaquet et al., 2014), while in text, shorter segments elicit stronger emotional reactions (Schindler et al., 2017), emphasizing the need for careful stimulus formatting.

## 7 Conclusion

Affective stimulus databases have advanced psychophysiological research by linking emotional experiences with physiological markers. However, challenges such as variability in methods, validation, and cultural specificity continue to impede progress. Addressing these issues through standardized protocols, technological innovation, and cross-cultural validation is critical for enhancing the robustness and replicability of future studies. As the field embraces emerging methods such as virtual reality and machine learning, concerted efforts to refine affective databases will pave the way for deeper insights into the complex interplay between emotion, cognition, and physiology.

## Glossary

IAPS: International Affective Picture System
IADS: International Affective Digitized Sounds
VR: Virtual Reality
KAPODI: Searchable Database of Emotional Stimuli Sets
DEVO: Database of Emotional Videos from Ottawa
EmoMadrid: Emotional Pictures Database for Affect Research
EPSS: Empathy for Pain Stimuli System
ESISCA: The Pictorial Set of Emotional Social Interactive Scenarios between Chinese Adults
SocialVidStim: Social Video Stimuli
luVRe: Library for Universal Virtual Reality Experiments
AVDOS-VR: Affective Video Database with Physiological Signals and Continuous Ratings Collected Remotely in VR
IAVRS: International Affective Virtual Reality System
GEMS: Geneva Emotional Music Scale
EmoMusic: Emotionally Labeled Music Database
MIAS: Mood Inductive Audio Stories.
EMOVO Corpus: Italian Emotional Speech Database
ANEW: Affective Norms for English Words
SAM: Self-Assessment Manikin
SCR: Skin Conductance Response
EEG: Electroencephalography
MEG: Magnetoencephalography
fMRI: Functional Magnetic Resonance Imaging
ERP: Event-Related Potentials
